# New perspectives in the genetic diagnosis of male infertility

**DOI:** 10.3325/cmj.2021.62.201

**Published:** 2021-06

**Authors:** Rossella Cannarella, Rosita A. Condorelli, Sandro La Vignera, Aldo E. Calogero

**Affiliations:** Department of Clinical and Experimental Medicine, University of Catania, Catania, Italy *aldo.calogero@unict.it*

The current issue of the *Croatian Medical Journal* features two interesting articles on reproductive health. Sengun et al (1) present their findings of novel mutations in the gene coding for FK506 binding protein-like (*FKBPL*) associated with male infertility, while Bilić et al (2) discuss the benefits of ovarian tissue cryopreservation. In spite of tremendous advances in the field, various aspects of reproductive health, particularly infertility, still necessitate further study and development of novel diagnostic and therapeutic approaches.

Infertility, defined by the World Health Organization as the failure to achieve conception after 12-24 months of unprotected sexual intercourse (3), affects about one out of six couples of childbearing age in Western countries (4). Male factor infertility is identified in about half of the cases (5) and most of the times presents as spermatogenesis failure (SPGF). Spermatogenesis is a 74-day-long process involving up to 2000 genes. Among them, 600 to 900 are exclusively expressed by the male germ-line (6-8). Despite a comprehensive diagnostic workup, in the majority of cases the etiology of SPGF remains elusive. A monocentric study on 1737 infertile patients reported idiopathic oligozoospermia in ~ 75% of cases (9). Classic genetic screening can identify the cause of male infertility in only 5% of unselected patients and in 20% of patients with non-obstructive azoospermia (NOA) (10). In the remaining cases, the etiology of infertility is elusive.

In recent years, several mutations of genes involved in different steps of spermatogenesis, such as spermatogonia proliferation or meiotic division of primary spermatocytes, have been reported capable of interfering with murine spermatogenesis (11). Interestingly, many of them have been validated in infertile patients, as shown in case reports or case series. Thus, the inclusion of a comprehensive gene panel in the diagnostic algorithm of male infertility could raise the diagnostic yields (11). The first study addressing this issue analyzed a panel of three genes: *NR5A1*, *DMRT1*, and *TEX11*, in a cohort of 80 patients with NOA. *NR5A1* (9q33.3) encodes for a transcription factor involved in the regulation of genes playing a role in steroidogenesis. Its mutation has been associated with a wide spectrum of phenotypes, ranging from 46,XY partial and complete gonadal dysgenesis, to hypospadias, micropenis, anorchia, and, in otherwise healthy men, oligozoospermia or NOA. *DMRT1* (9p24.3) encodes for a testis-specific transcription factor acting in testis differentiation, whose mutations have been reported in patients with NOA. Finally, *TEX11* (Xq13.1) encodes for a meiosis-specific factor, involved in double-strand breaks DNA repair, which may play a role in NOA pathogenesis due to meiotic arrest (11). Out of 80 patients with NOA investigated for *NR5A1*, *DMRT1*, and *TEX11* gene mutations, the authors reported pathogenic variants in four, thus raising the diagnostic yield by 25% (10). A subsequent study has developed a gene panel including 15 genes (Table 1) involved in germ-cell proliferation and meiotic division. Interestingly, pathogenic mutations of *NR5A1* and *TEX11* genes were reported in 3/25 patients, increasing the diagnostic rate by 12%. Noteworthy, 11 likely pathogenic variants meriting functional analysis or segregation studies were also observed.

**Table 1:** Genes whose mutations cause spermatogenic failure characterized by a decreased sperm number (12)*

Gene	Inheritance	OMIM number	OMIM phenotype	Spermatogenic defect	Mutation detection frequency	HGNC gene number
*NR5A1*	AR	184757	SF8	Azoospermia Oligozoospermia	2% (7/315)	Nuclear receptor subfamily 5, group A, member 1
*SYCP3*	AD	604759	SF4	Azoospermia Oligozoospermia	10.5% (2/19)	Synaptonemal complex protein 3
*ZMYND15*	AR	614312	SF14	Azoospermia Oligozoospermia	1 consanguineous family	Zinc finger, MYND-type containing 15
*TAF4B*	AR	601689	SF13	Azoospermia Oligozoospermia	1 consanguineous family	TAF4b RNA polymerase II, TATA box binding protein (TBP)-associated factor
*TEX11*	XLR	300311	SF, X-linked, 2	Azoospermia	1%-2.4% (7/289) azoospermia; 15% azoospermia with meiotic arrest	Testis expressed 11
*NANOS1*	AD	608226	SF12	Azoospermia Oligozoospermia OAT	2.6% (5/195)	Nanos homologue 1 (Drosophila)
*PLK4*	AD	605031	-	Azoospermia (Sertoli cell-only syndrome)	1.2% (1/81)	Polo like kinase 4
*MEIOB*	AR	617670	SF22	NOA	1 consanguineous family	Meiosis specific with OB domains
*SYCE1*	AR	611486	SF15	NOA	1 consanguineous family	Synaptonemal complex central element protein 1
*USP9Y*	YL	400005	SF, Y-linked, 2	NOA	3 probands (4-db DEL; DEL incl. entire gene)	Ubiquitin specific peptidase 9, Y-linked
*SOHLH1*	-	610224	-	NOA	2% (2/100)	Spermatogenesis and oogenesis specific basic helix-loop-helix 1
*RHOXF2*	-	300447	-	Severe oligozoospermia	<1% (1/250)	Rhox homeobox family member 2
*TEX15*	AR	605795	-	Azoospermia Oligozoospermia	2 family; 1 proband	Testis expressed 15, meiosis and synapsis associated
*HSF2*	AD	140581	-	Azoospermia	<1%(1/766)	Heat shock transcription factor 2
*KLHL10*	AD	608778	SF11	OAT	1.3% (7/556)	Kelch-like family member 10

*Abbreviations: OMIM – Online Mendelian Inheritance in Man**;** HGNC – Hugo Gene Nomenclature Committee; AD – autosomal dominant; AR – autosomal recessive; NOA – non-obstructive azoospermia; OAT – oligo-astheno-teratozoospermia; SF – spermatogenic failure.

These data highlight the importance of designing a comprehensive and accurate gene panel to be used in patients with otherwise unexplained SPGF. Furthermore, next-generation sequencing makes this analysis widely accessible from both an economic and a geographic point of view. An appropriate gene panel for SPGF could facilitate the identification of the genetic cause of infertility (when present), but in the future it may also represent a diagnostic test predicting sperm recovery after testicular sperm extraction (TESE). Accordingly, a study matching the results of testicular histology with those of genetic testing has been published and others are currently ongoing. Interestingly, mutations (eg, deletions, missense, stop-gain) of specific genes involved in meiosis (eg, *M1AP*, *ADAD2*, *TERB1*, *SHOC1*, *MSH4*, *RAD21L1*, *TEX14*, *DMRT1*, *TEX11*, *SYCE1*, *MEIOB*, *MEI1*, *STAG3-a*) have been reported in patients with meiotic arrest (13,14). If validated, these gene variations may be included in a pre-TESE prognostic gene panel, which may help to determine the chance of sperm recovery. Finally, another challenge in this field is to understand the implications of the transmission of these gene mutations to the offspring.

In conclusion, the evidence favoring the inclusion of SPGF monogenic mutation assessment in the diagnostic workup of male infertility is accumulating. Despite this, a comprehensive panel has not yet been validated, although some gene mutations are more frequently present than others (eg, *NR5A1* or *TEX11*), and others have recently been discovered. Since more than 2000 genes are involved in spermatogenesis, we are still far from a comprehensive view of the monogenic etiology of SPGF. However, in the near future, this evidence is likely to practically affect the diagnostic workup and decision-making algorithms of male infertility.
